# Eating attitudes across body mass index categories in Saudi Arabia: a cross-sectional study

**DOI:** 10.3389/fnut.2026.1737215

**Published:** 2026-02-12

**Authors:** Hayat Alzahrani, Manal Naseeb, Alyaa M. Zagzoog, Sundus Malaikah, Soaad Alsulami, Eram Albajri

**Affiliations:** 1 Department of Food Science and Nutrition, College of Food and Agricultural Sciences, King Saud University, Riyadh, Saudi Arabia; 2 Department of Clinical Nutrition, Faculty of Applied Medical Sciences, King Abdulaziz University, Jeddah, Saudi Arabia; 3 Department of Community Health, Faculty of Applied Medical Sciences, Northern Border University, Arar, Saudi Arabia

**Keywords:** behavior, eating attitude, eating disorders, food preoccupation, obesity

## Abstract

**Introduction:**

Body weight is linked to disordered eating through psychological, behavioral, and sociocultural mechanisms. Studies in Saudi Arabia reported increased eating disorder risk, particularly among female university students; however, associations between body mass index (BMI) categories and specific EAT-26 subscales remain underexplored. This study assessed eating attitudes among Saudi adults with different BMI categories, examining associations with BMI, and identifying sociodemographic and metabolic risk factors.

**Methods:**

This cross-sectional study surveyed Saudi adults (≥18 years) using convenience sampling via social media between May 30, 2021, and May 22, 2024. Body mass index (BMI) categories were defined as overweight (25.0–29.9 kg/m^2^) and obesity (≥30 kg/m^2^). Eating attitudes were assessed using the validated Arabic EAT-26. Multinomial logistic regression examined associations between eating disorder status and BMI categories with sequential adjustment for demographic and health-related factors. Linear and multinomial regression models evaluated relationships between EAT-26 subscales and BMI as continuous and categorical outcomes using SPSS version 27.

**Results:**

Among 404 Saudi adults, mean BMI was higher in males than females (26.5 ± 5.4 vs. 24.7 ± 5.5 kg·m^−2^, *p* < 0.001). In age- and sex-adjusted multinomial logistic regression, participants with a possible eating disorder had a higher relative risk of being overweight compared with normal weight (RRR = 5.90, 95% CI: 1.30–26.83). In linear regression analyses, higher scores for others' perception and oral control were inversely associated with across the full BMI distribution.

**Discussion/conclusion:**

These findings underscore the influence of sociocultural change in Saudi Arabia on eating attitudes, where Westernized body ideals and evolving social expectations shape body image concerns. Eating-related attitudes, particularly among individuals in the overweight range, may reflect heightened social sensitivity and self-regulation rather than pathological disordered eating. Culturally sensitive psychosocial screening and stigma-reducing, body-respectful public health strategies may support psychological wellbeing and healthier weight trajectories.

## Introduction

In Saudi Arabia, the prevalence of obesity has increased to 35.6%, compared to the global rate (1). The World Health Organization defines obesity as a body mass index (BMI) of 30 kg/m^2^ or higher, while a BMI of 25.0–29.9 kg/m^2^ is classified as overweigh (2). Obesity can increase the risk of other co-morbidities, such as heart disease and type 2 diabetes mellitus (2, 3). Beyond cardiometabolic consequences, weight status may be associated with eating-related concerns that influence nutrition and wellbeing. Excess body weight and obesity may be associated with disordered eating attitudes and behaviors, which represent an important and often under-recognized dimension of nutrition- and weight-related health (4).

Accordingly, examining eating attitudes across BMI categories provides important insight into the psychosocial dimensions of excess body weight beyond its metabolic consequences. Understanding how eating attitudes vary by BMI, alongside sociodemographic and health-related factors, may help identify groups at increased risk for maladaptive eating-related concerns. Such evidence is essential to inform targeted, culturally appropriate prevention and intervention strategies that address both nutritional health and psychological wellbeing among Saudi adults.

Eating attitudes such as excessive preoccupation with food, restrictive dieting, fear of weight gain, or compensatory behaviors may occur across the weight spectrum and can influence dietary quality, psychological wellbeing, and health behaviors. The Eating Attitudes Test (EAT-26) is a widely used self-report screening tool designed to identify individuals with elevated risk of disordered eating based on attitudes, feelings, and behaviors related to eating (5, 6). It involves three subscales: dieting, bulimia and food preoccupation, and oral control, which provide a structured assessment of eating-related concerns commonly associated with disordered eating risk (5). In Saudi Arabia, the Arabic EAT-26 was validated in a large non-clinical Saudi sample, which supported its use for assessing disordered eating attitudes and behaviors in Saudi populations (7). Given its established validity within the Saudi context, the Arabic EAT-26 provides a suitable framework for examining eating attitudes across different BMI categories in this population. Applying this tool allows for the assessment of how specific eating attitude subscales relate to body weight status and sociodemographic characteristics.

Research has linked excess body weight to the risk of eating disorders and disordered eating through a complex interplay of psychological, behavioral, and sociocultural factors (8). For example, in Saudi Arabia, the disordered eating attitude score was higher in females university members with obesity compared to other BMI categories (9). Additionally, among Saudi female university students, a notable prevalence and associations with body image concerns, dieting behaviors, and psychosocial risk factors were observed (10). Using the EAT-26 scale, more than one-fourth of the participating female students at Jazan University were at risk for eating disorders (EDs), in particular bulimia nervosa and binge eating disorder (11). An eating disorder (ED) is a mental health condition involving unhealthy eating behaviors and concerns about food, weight, or body shape that affect physical and psychological wellbeing (8). BMI was associated with the risk of EDs in this population (11).

Recent studies in Saudi Arabia highlight notable regional and population-level variations in the prevalence of disordered eating and its association with body weight and lifestyle factors.

A systematic review found that the prevalence of EDs and disordered eating behaviors in the eastern region of Saudi Arabia was higher among older school students compared to other regions (12). However, the relationship between different BMI categories and EAT-26 subscales; specifically dieting, bulimia and food preoccupation, and oral control, needs further investigation. Another study found that obesity among Saudi medical students was associated with a higher risk of disordered eating behaviors, based on validated screening tools (13). Interestingly, a considerable prevalence of disordered eating, with significant associations with gender, body mass index, and lifestyle factors are found among Saudi Electronic University students (14). These findings suggest the need for further research to better understand eating attitudes across BMI categories and their potential implications for prevention and support strategies within Saudi settings.

Understanding and addressing barriers to healthy eating among adults, while considering cultural factors, is an important area for investigation (15). Awareness is another important factor to consider, as Alhelal and Tami (16) assessed Saudi adults' awareness of caloric intake and weight management, and identified gaps that may contribute to unhealthy eating behaviors and obesity. Additionally, a recent cross-sectional study in Jeddah found that over half of adolescent girls scored at or above the EAT-26 cutoff, and that social media negatively influenced eating attitudes among approximately half of participants (17, 18). However, the relationship between BMI categories and the subscales of eating attitudes in the Saudi Arabian context needs further investigation. Addressing this gap is essential to better understand how eating attitudes vary across weight categories within the Saudi population. Examining these relationships may help identify early psychosocial risk factors and inform culturally sensitive strategies to promote healthier eating behaviors and weight management.

Accordingly, we hypothesized that individuals with obesity would demonstrate higher scores on disordered eating subscales, particularly anorexic and bulimic attitudes, compared with participants with normal body weight. This study aimed to (1) determine the prevalence of EDs among BMI categories in Saudi adults; (2) evaluate the relationship between BMI, and eating attitude; and (3) identify sociodemographic and health-related factors associated with eating attitudes in the study population, including variables such as age, gender, education level, social status, ED status, and metabolic disorders.

## Materials and methods

### Study design

This cross-sectional study employed an online survey to identify the presence of eating disorder risk based on attitudes, feelings, and behaviors related to eating among adults residing in Saudi Arabia. The study was reviewed and approved by the Research and Ethics Committee of King Abdulaziz University Hospital (KAUH). The Institutional Review Board (IRB) determined that the study was exempt from full review (Reference No. 2-22), as it involved the use of de-identified survey data and posed no risk to participants. Participants provided informed consent prior to engaging in this study.

### Participant recruitment

Eligible participants included adults aged 18 years and older. The exclusion criteria were women who were pregnant or lactating. Participants were recruited via convenience sampling through social media platforms, such as X (formerly known as Twitter), WhatsApp, word of mouth, and the King Abdulaziz University (KAU) internal email network. Data collection spanned 3 years, from May 30, 2021, to May 22, 2024. The extended collection period was necessitated primarily by recruitment challenges, particularly in enrolling male participants.

### Data collection tools

This study used the Arabic version of the Eating Attitudes Test-26 (EAT-26) to screen for symptoms and concerns related to eating disorders anorexia nervosa, bulimia nervosa and binge eating disorder (5, 7). The EAT-26 employs a 6-point Likert scale ranging from “always” to “never,” allowing respondents to indicate the frequency of specific behaviors and thoughts associated with disordered eating. The scale has three subscales: dieting (items 1, 6, 7, 10, 11, 12, 14, 16, 17, 22, 23, 24, 26), bulimia and food preoccupation (items 3, 4, 9, 18, 21, 25), and oral control (items 2, 5, 8, 13, 15, 19, 20). The Arabic version of the EAT-26 retains the original three-subscale structure (Dieting, Bulimia and Food Preoccupation, and Oral Control); however, validation studies have additionally examined culturally relevant response patterns and item-level behaviors to ensure appropriateness within Arabic-speaking populations (7). The scoring system ranges from 0 to 78, with higher scores indicating greater concern regarding eating attitudes. A score of 20 or above is considered indicative of is defined as being characteristic of a “disordered eating attitude” Scores on the three subscales can be examined to determine the focus of disordered eating. In addition to the raw scores, the results are presented as two percentiles based on published normative data (5, 6).

### Statistical analysis

Statistical analyses were performed using SPSS software package v27. Normality of variables was assessed using histograms and Shapiro-Wilk test. Due to non-normal distributions, age and BMI were log-transformed for inferential analyses. Continuous variables were summarized as means and standard deviations (SD) for normally disturbed data, median and interquartile range (IQR) for non-normally distributed data, and frequencies and percentages for categorical variables. Sex differences in participant characteristics were assessed using independent samples *t*-tests for normally distributed variables, Mann–Whitney U for non-normally distributed variables, and chi-square (χ^2^) tests for categorical variables. To examine the association between eating disorder status and BMI categories, multinomial logistic regression models were fitted with BMI category as the outcome variable. and possible eating disorder status (yes vs. no) as the exposure. Model 1 was adjusted for age and sex; Model 2 was further adjusted for monthly income and education level; and Model 3 was additionally adjusted for medication use. Unless otherwise specified, all regression models followed the same sequence of adjustment. Data are presented as relative risk ratios (RRRs) with 95% confidence intervals (CI).

Associations between eating attitude subscales and BMI were examined using linear regression models with BMI treated as a continuous outcome (presented in the Supplementary Table 1). To examine associations between eating attitude subscales and BMI categories, multinomial logistic regression analyses were conducted RRRs with 95% CIs were estimated per one-unit increase in eating attitude subscale scores (presented in the Supplementary Table 2). Subgroup analyses were conducted to examine associations between the impact of others' perception subscale and continuous BMI across age, sex, and income categories. Linear regression models were used within each subgroup. All subgroup models were adjusted for age and sex, except for analyses stratified by age or sex, in which the corresponding variable was excluded from the model. All statistical tests were two-sided, and a *p*-value < 0.05 was considered statistically significant.

## Results

### Participants characteristics

A total of 404 participants were included in the analysis, of whom 234 (57.9%) were female and 170 (42.1%) were male (Table 1). The median BMI of the total sample was 24.65 (6.78) kg/m^2^. Median BMI was significantly higher in males than in females [25.89 (6.71) vs. 24.08 (6.73) kg/m^2^, *p* < 0.001]. Males were also older than females, with a higher median age [33 (18) vs. 29 (8) years, *p* = 0.010]. Significant sex differences were observed across geographic region, educational level, occupation, and monthly income (*p* < 0.001). In this sample, a higher proportion of the females resided in the Western region, whereas males were more evenly distributed across regions. Educational attainment differed by sex, with a greater proportion of females holding bachelor's or postgraduate degrees. Employment patterns also differed, with males more frequently employed in government or private sectors, while females were more commonly unemployed or categorized as “other.” Overall, 45.3% of participants were classified as normal weight, 29.5% as overweight, 18.1% as obese, and 7.2% as underweight (Table 1). BMI category distribution differed significantly by sex (*p* < 0.001). Underweight and normal weight classifications were more common among females, whereas overweight and obesity were more prevalent among males.

**Table 1:** Participants characteristics.

**Variable**	**Overall *n* = 404**	**Female *n* = 234**	**Male *n* = 170**	***p*-value**
Age (years)	30.50 (18)	29 (18)	33 (18)	**0.010**
BMI (kg·m^−2^)	24.65 (6.78)	24.08 (6.73)	25.89 (6.71)	**< 0.001**
**Region**				<**0.001**
North region	24 (5.9%)	7 (3.0%)	17 (10%)	
South region	38 (9.4%)	9 (3.8%)	29 (17%)	
East region	30 (7.4%)	9 (3.8%)	21 (12%)	
Central region	33 (8.2%)	12 (5.1%)	21 (12%)	
West region	279 (69%)	197 (84%)	82 (48%)	
**Educational level**				<**0.001**
High school and lower	45 (11%)	19 (8.1%)	26 (15%)	
Diploma	32 (7.9%)	9 (3.8%)	23 (14%)	
Bachelor's degree	209 (52%)	132 (56%)	77 (45%)	
Post-graduate degree	118 (29%)	74 (32%)	44 (26%)	
**Occupation**				<**0.001**
Government sector (health or non-health)	163 (40%)	77 (33%)	86 (51%)	
Private sector or Business	66 (16%)	27 (12%)	39 (23%)	
Non-profit sector	8 (2.0%)	1 (0.4%)	7 (4.1%)	
Unemployed or Others	167 (41%)	129 (55%)	38 (22%)	
**Monthly income**				<**0.001**
< 5,000 SAR	147 (36%)	107 (46%)	40 (24%)	
5,000–15,000 SAR	137 (34%)	77 (33%)	60 (35%)	
16,000–30,000 SAR	81 (20%)	40 (17%)	41 (24%)	
>30,000 SAR	39 (9.7%)	10 (4.3%)	29 (17%)	
**Marital status**				**0.079**
Married	174 (43%)	94 (40%)	80 (47%)	
Single	199 (49%)	124 (53%)	75 (44%)	
Divorced	28 (6.9%)	16 (6.8%)	12 (7.1%)	
Widower	3 (0.7%)	0 (0%)	3 (1.8%)	
**BMI classification**				<**0.001**
Underweight (< 18.5 kg·m^−2^)	29 (7.2%)	25 (11%)	4 (2.4%)	
Normal (18.5–24.9 kg·m^−2^)	183 (45%)	116 (50%)	67 (39%)	
Overweight (25–29.9 kg·m^−2^)	119 (29%)	55 (24%)	64 (38%)	
Obese (≥30 kg·m^−2^)	73 (18%)	38 (16%)	35 (21%)	

Data are presented as median and interquartile range or frequencies (%). Differences between sexes were assessed using the Wilcoxon rank-sum test for continuous variables and the chi-square for categorical variables. Age and BMI were analyzed using log-transformed values due to non-normal distributions, with untransformed values shown for clarity. p < 0.05 was considered statistically significant.

### Eating disorder status and BMI categories

In age and sex adjusted multinomial logistic regression analyses, participants with a possible eating disorder had a higher relative risk of being classified as overweight rather than normal weight (RRR = 5.90, 95% CI: 1.30–26.83, *p* = 0.022) (Table 2). The relative risk of obesity compared with normal weight was higher among participants with a possible eating disorder; however, this association did not reach statistical significance (RRR = 3.74, 95% CI: 0.82–17.03, *p* = 0.089). No significant association was observed between eating disorder status and underweight classification (RRR = 2.02, 95% CI: 0.42–9.64, *p* = 0.377).

**Table 2:** Association between eating disorder status and BMI categories using multinomial logistic regression.

**BMI category vs. normal weight**	**Model 1 RRRs (95% CI)**	***p*-value**	**Model 2 RRRs (95% CI)**	***p*-value**	**Model 3 RRRs (95% CI)**	***p*-value**
Obese	3.74 (0.82–17.03)	0.089	4.55 (0.96–21.61)	0.056	4.57 (0.96–21.74)	0.056
Overweight	5.90 (1.30–26.83)	**0.022**	6.29 (1.36–29.03)	**0.018**	6.29 (1.36–29.08)	**0.018**
Underweight	2.02 (0.42–9.64)	0.377	2.09 (0.43–10.22)	0.363	2.07 (0.42–10.12)	0.371

Data are presented as relative risk ratios (RRRs) and 95% confidence intervals were estimated using multinomial logistic regression models. Normal weight was the outcome reference category, and no possible eating disorder was the exposure reference category. Model 1 adjusted for age and sex, model 2 further adjusted for monthly income and education level, model 3 additionally adjusted for medication use. Bold values indicate statistical significance at *p* < 0.05.

### Associations between eating attitude subscales and BMI

In linear regression analyses adjusted for age and sex, higher scores on the *impact of others' perception* (β = −0.287, *p* < 0.001) subscale was inversely associated with BMI across the full BMI distribution (Table 3). These associations remained statistically significant in models further adjusted for monthly income and education level (Model 2) and additionally, for medication use (Model 3), as presented in Supplementary Table 1. No statistically significant associations were observed for the remaining eating attitude subscales.

**Table 3:** Associations between eating attitudes subscales and BMI.

**Eat subscales**	**β (95% CI)**	***p*-value**
1. Restrained eating attitude	0.015 (−0.083 to 0.114)	0.759
2. Anorexic attitudes	−0.055 (−0.147 to 0.037)	0.238
3 & 4. Cycle of bulimic crises	0.073 (−0.007 to 0.153)	0.072
5. Impact of others' perception	−0.287 (−0.454 to −0.121)	**< 0.001**
6. Choice of food quality and time spent on meals	−0.150 (−0.362 to 0.062)	0.166

Data are presented as beta coefficients (β) and 95% confidence intervals derived from linear regression models. Body mass index (BMI) was modeled as a continuous outcome. Models were adjusted for age and sex. Beta coefficients represent the mean change in BMI per one-unit increase in the eating attitude subscale score. Bold values indicate statistical significance at *p* < 0.05.

### Associations between eating attitude subscales and BMI categories

In multinomial logistic regression analyses examining associations between eating attitude subscales and BMI categories, with normal weight as the reference category (Table 4), higher scores on the *impact of others' perception* subscale were associated with a lower relative risk of obesity compared with normal weight in the age and sex adjusted model (RRR = 0.80, 95% CI: 0.65–0.99, *p* = 0.040). No other eating attitude subscales were significantly associated with obesity, overweight, or underweight in this model.

**Table 4:** Associations between eating attitudes subscales and BMI categories.

**Eating subscale**	**Obese vs. normal RRR (95% Cl)**	***p*-value**	**Overweight vs. normal RRR (95% Cl)**	***p*-value**	**Underweight vs. normal RRR (95% Cl)**	***p*-value**
**Model 1**						
1. Restrained eating attitude	0.96 (0.83–1.11)	0.553	0.92 (0.81–1.04)	0.164	0.99 (0.79–1.24)	0.943
2. Anorexic attitudes	0.97 (0.80–1.17)	0.754	0.97 (0.83–1.14)	0.749	1.11 (0.84–1.48)	0.467
3 & 4. Cycle of bulimic crises	1.06 (0.90–1.26)	0.484	1.06 (0.92–1.23)	0.400	1.25 (0.96–1.64)	0.102
5. Impact of others' perception	0.80 (0.65–0.99)	**0.040**	0.87 (0.72–1.04)	0.125	0.95 (0.70–1.31)	0.765
6. Choice of food quality and time spent on meals	0.97 (0.83–1.13)	0.669	0.97 (0.85–1.10)	0.593	1.03 (0.84–1.27)	0.756

Data are presented as relative risk ratios (RRRs) with 95% confidence intervals estimated using multinomial logistic regression. Normal weight was the outcome reference category. Model 1 was adjusted for age and sex. RRRs represent the relative risk of each BMI category per one-unit increase in the eating attitude subscale score. Bold values indicate statistical significance at *p* < 0.05.

In models further adjusted for monthly income and education level (Model 2; Supplementary Table 2), the association between the *impact of others' perception* subscale and obesity was attenuated and did not reach statistical significance (RRR = 0.82, 95% CI: 0.66–1.02, *p* = 0.070). In this model, higher scores on the *bulimia and food preoccupation* subscale were associated with a lower relative risk of underweight compared with normal weight (RRR = 0.69, 95% CI: 0.47–1.00, *p* = 0.049).

In the fully adjusted model including medication use (Model 3; Supplementary Table 2), the inverse association between *bulimia and food preoccupation* and underweight remained statistically significant (RRR = 0.68, 95% CI: 0.47–0.99, *p* = 0.045). No other eating attitude subscales were significantly associated with BMI categories across Models 2 and 3, and no consistent associations were observed for restrained eating or anorexic attitudes.

### Subgroup analyses by age, sex, and income

Subgroup analyses examined associations between the *impact of others' perception* subscale and BMI across age, sex, and income categories (Table 5). For the *impact of others' perception*, inverse associations with BMI were observed in early adulthood (30–39 years; β = −0.65, 95% CI: −1.21 to −0.08, *p* = 0.028) and were of borderline significance in young adulthood (18–29 years; *p* = 0.050). No statistically significant associations were observed in middle or older age groups. Sex-stratified analyses showed a borderline inverse association between the *impact of others' perception* and BMI among females (*p* = 0.055), whereas no association was observed among males. No significant associations were identified across income strata.

**Table 5:** Subgroup analyses of the association between the impact of others' perception subscale and BMI by age, sex, and income.

**Age group**	**β (95% CI)**	***p*-value**
Young adulthood (18–29 years)	−0.35 (−0.71 to 0.00)	**0.050**
Early adulthood (30–39 years)	−0.65 (−1.21 to −0.08)	**0.028**
Middle adulthood (40–49 years)	−0.17 (−0.56 to 0.21)	0.373
Late middle adulthood (50–59 years)	−0.90 (−1.97 to 0.17)	0.087
Older adults (≥60 years)	−0.15 (−0.40 to 0.09)	0.220
**Sex**		
Female	0.01 (−0.53 to 0.55)	**0.055**
Male	−0.16 (−0.49 to 0.16)	0.969
**Monthly income**		
< 5,000 SAR	0.11 (−0.25 to 0.48)	0.324
5,000–15,000	−0.16 (−1.66 to 1.33)	0.204
16,000–30,000 SAR	−0.35 (−0.71 to 0.00)	0.516
>30,000 SAR	−0.65 (−1.21 to −0.08)	0.799

Data are presented as beta coefficients (β) with 95% confidence intervals derived from linear regression models examining the association between the impact of others' perception subscale and continuous BMI. Models were adjusted for age and sex, except for analyses stratified by age or sex, where the corresponding variable was not included in the model. Bold values indicate statistical significance at *p* < 0.05.

## Discussion

In our study, we found that eating disorder risk status showed a selective association with BMI category among Saudi adults. Relative to participants with no possible eating disorder, those with a possible eating disorder status had a significantly higher relative risk of being overweight compared with normal weight, and this association remained consistent across multinomial models accounting for age, sex, socioeconomic factors, and medication use. In contrast, the association with obesity did not reach conventional statistical significance, although the direction and magnitude suggested a borderline trend. No meaningful association was observed for underweight category. When disordered eating attitudes were examined dimensionally, most EAT subscales were not independently associated with BMI after adjustment. Two domains demonstrated the most consistent relationships. The impact of others' perception was inversely associated with BMI across models, indicating that social evaluative concerns and self-regulatory aspects of eating were the attitudes most closely linked to weight status in this population.

These findings suggest that psychosocial and social evaluative dimensions of eating, rather than classical behavioral or pathological eating disorder constructs, are most relevant to BMI differences in this sample. The prominence of the impact of others' perception indicates that how individuals experience social scrutiny, internalize external expectations, and regulate their eating behavior may play a more important role than overt restrictive, bulimic, or dieting behaviors captured by traditional EAT subscales. This pattern aligns with psychosocial models that emphasize the influence of social norms, appearance-related expectations, and internalized evaluation on eating-related cognition and behavior, particularly in societies undergoing rapid cultural and lifestyle transitions (19, 20). The inverse association between the impact of others' perception and BMI suggests that individuals who are more sensitive to social judgment surrounding eating may engage in greater self-monitoring of food intake, which could function as a protective factor against higher BMI (21, 22). Prior research has shown that social evaluation and perceived surveillance can influence eating behavior in complex ways, acting either as a source of distress or as a regulatory mechanism depending on how such pressures are internalized (20, 23). This social evaluative dimension appears more closely aligned with regulation than with pathology.

The absence of independent associations for other EAT subscales, including anorexic attitudes, dieting behaviors, and bulimic tendencies, further reinforces the conclusion that BMI differences in this population are not primarily driven by classical eating disorder psychopathology. Instead, the findings may point to a more nuanced psychosocial pathway in which social perception, self-evaluation, and regulatory attitudes toward eating play central roles. This perspective may also help explain why eating disorder risk status was most strongly associated with being overweight rather than obese. Individuals in the overweight range may remain closer to socially defined norms of acceptable body size and therefore be more responsive to social comparison and perceived scrutiny. In contrast, obesity is likely characterized by greater heterogeneity in biological, psychological, and social influences that may attenuate the observable impact of eating-related attitudes alone (24, 25).

Clear differentiation was also observed across BMI categories. The absence of a clear association with underweight may indicate that the EAT constructs examined in this study are not equally related to all departures from normal weight (26). Rather than reflecting a linear relationship across the BMI spectrum, the findings indicate a category-specific pattern in which overweight occupies a distinct psychosocial position (26). Individuals in this category may experience heightened body awareness and social evaluation without the degree of metabolic or health-related burden often associated with obesity, making eating-related attitudes more influential at this stage of weight status (27).

Subgroup analyses provided further insight into potential differences in associations across age and sex groups; however, these results should be interpreted with caution due to limited statistical power within strata. The negative association between the impact of others' perceptions and BMI was more pronounced among younger individuals and those in early adulthood, suggesting that perceived social evaluation and external judgment may play a greater role in shaping eating-related attitudes during earlier stages of adulthood (28). This pattern aligns with developmental evidence showing that appearance concerns, social comparison, and peer acceptance are especially influential during early life stages when self-concept is shaped by social feedback (29, 30). Sex-stratified analyses indicated a borderline inverse association among females but not males, potentially reflecting sex differences in body image norms and societal expectations related to eating and appearance (31, 32). Nevertheless, the exploratory nature of these analyses and the lack of consistent statistical significance underscore the need for cautious interpretation.

These subgroup patterns may be understood within the broader sociocultural context of Saudi Arabia, where rapid social change and increasing exposure to Western media and beauty standards have altered perceptions of body image and eating behavior (33, 34). As traditional cultural values intersect with contemporary ideals of appearance, individuals may experience tension between established norms and emerging expectations (35). Such cultural dissonance may be particularly relevant for adults in the overweight range, who may perceive themselves as misaligned with shifting social standards and therefore be more sensitive to external evaluation (36). Heightened attention to eating and body-related concerns may reflect efforts to negotiate social conformity rather than the presence of classical eating disorder psychopathology (32, 37).

The importance of cultural context is further underscored by evidence that Westernized body ideals, media exposure, and diet-related norms are increasingly shaping eating-related attitudes across Middle Eastern populations (38). These transitions may intensify social comparison processes and internalized appearance standards, leading eating-related attitudes to be driven more strongly by perceived social scrutiny and self-regulation than by overt disordered eating behaviors. Greater sensitivity to social judgment and stronger regulatory control overeating may function as adaptive responses to evolving social expectations rather than inherently pathological traits (39). These findings suggest that body-respectful messaging and culturally sensitive nutrition counseling may be especially relevant in contexts of heightened weight-related social evaluation.

The results indicate that psychosocial factors related to eating may be more pronounced during the early phases of weight gain, particularly among individuals classified as overweight, suggesting the importance of early detection and preventive efforts that go beyond clinically apparent eating disorder symptoms. Incorporating brief psychosocial screening measures into routine weight-related evaluations may assist in identifying individuals who could benefit from timely, culturally sensitive support before biological or medical complications emerge. At the population level, public health strategies that address weight-related stigma and foster supportive social environments—especially for younger adults and women—may help promote healthier weight outcomes within the Saudi context.

This study includes several strengths that support the clarity and interpretation of its findings. Notably, the use of a validated Arabic version of the EAT-26 ensured linguistic and cultural relevance when assessing eating attitudes within a Saudi population. Nonetheless, several limitations should be considered. The cross-sectional design limits the ability to draw conclusions regarding causality or temporal relationships. Reliance on self-reported data may introduce reporting or social desirability bias, particularly in contexts where disordered eating may be stigmatized or underrecognized. Additionally, online recruitment may have contributed to selection bias, as participants with higher health awareness or interest in nutrition-related topics may have been more likely to participate, potentially affecting generalizability. The observed sex imbalance in the sample may also have influenced the findings, given established sex differences in eating attitudes and body image concerns. Finally, while subgroup analyses offered exploratory insights, smaller subgroup sizes may have limited statistical power, and findings should therefore be interpreted with caution.

## Conclusion

In a region where overweight and obesity continue to pose significant public health challenges, this study provides important insight into the psychosocial dimensions associated with BMI among Saudi adults. These findings demonstrate that eating disorder risk is selectively associated with overweight status, while obesity shows only a borderline association, and underweight shows no meaningful relationship. Moreover, the results indicate that most classical eating disorder attitudes are not independently linked to BMI, with social evaluative concerns emerging as the most consistent correlates of weight status. These patterns suggest that eating-related attitudes in this population reflect broader psychosocial and self-regulatory processes rather than disordered overeating psychopathology. Acknowledging the influence of social evaluation and behavioral regulation on weight status may support the development of more refined and culturally responsive strategies for screening, prevention, and intervention that extend beyond weight-focused frameworks and attend to the psychological contexts shaping eating behaviors.

## Data Availability

The raw data supporting the conclusions of this article will be made available by the authors, without undue reservation.
