# Supplementary material for: Eating attitudes across body mass index categories in Saudi Arabia: a cross-sectional study
Source: Front Nutr. 2026 Feb 12;13:1737215. doi: 10.3389/fnut.2026.1737215 (PMC12938680; doi:10.3389/fnut.2026.1737215)
Supplement: Supplementary file 1 [file Table_1.docx]

**Supplementary Materials**

**Table 1. Associations between eating attitudes subscales and BMI**

| **Eating subscale** | **Obese vs Normal**  **RRR (95% Cl)** | ***p-*value** | **Overweight vs Normal**  **RRR (95% Cl)** | ***p-*value** | **Underweight vs Normal**  **RRR (95% Cl)** | ***p-*value** |
| --- | --- | --- | --- | --- | --- | --- |
| **Model 2** | | | | | | |
| 1. Restrained eating attitude | 0.972 (0.838­–1.127) | 0.704 | 0.91 (0.80–1.03) | 0.149 | 0.96 (0.77-1.21) | 0.756 |
| 2. Anorexic attitudes | 0.980 (0.810-1.186) | 0.836 | 0.97 (0.82-1.14) | 0.684 | 1.07 (0.80-1.44) | 0.645 |
| 3&4. Cycle of bulimic crises | 1.089 (0.915-1.297) | 0.335 | 1.05 (0.90-1.22) | 0.534 | 1.28 (0.96-1.70) | 0.088 |
| 5. Impact of others’ perception | 0.818 (0.658-1.016) | 0.07 | 0.86 (0.71-1.03) | 0.106 | 0.95 (0.69-1.32) | 0.771 |
| 6. Choice of food quality and time spent on meals | 0.976 (0.837-1.138) | 0.755 | 0.97 (0.84-1.09) | 0.542 | 1.05 (0.85-1.29) | 0.656 |
| **Model 3** | | | | | | |
| 1. Restrained eating attitude | 0.97 (0.84–1.13) | 0.714 | 0.91 (0.80–1.03) | 0.150 | 0.97 (0.77–1.22) | 0.972 |
| 2. Anorexic attitudes | 0.98 (0.81–1.19) | 0.829 | 0.97 (0.82–1.14) | 0.682 | 1.08 (0.80–1.45) | 0.631 |
| 3&4. Cycle of bulimic crises | 1.09 (0.92–1.30) | 0.338 | 1.05 (0.90–1.22) | 0.537 | 1.28 (0.96–1.71) | 0.087 |
| 5. Impact of others’ perception | 0.82 (0.66–1.02) | 0.071 | 0.86 (0.71–1.03) | 0.106 | 0.96 (0.69–1.33) | 0.794 |
| 6. Choice of food quality and time spent on meals | 0.98 (0.84–1.14) | 0.752 | 0.96 (0.84–1.09) | 0.542 | 1.06 (0.86–1.32) | 0.576 |

Data are presented as relative risk ratios (RRRs; Exp[B]) with 95% confidence intervals estimated using multinomial logistic regression. Normal weight was the reference BMI category. RRRs represent the relative risk of each BMI category per one-unit increase in eating attitude subscale score. Model 2 was adjusted for age, sex, monthly income, and education level. Model 3 was additionally adjusted for medication use.

**Table 2. Associations between eating attitudes subscales and BMI categories**

| **Eating attitudes subscales** | **Mean ± SD** | **β (95% CI)** | ***p-*value** |
| --- | --- | --- | --- |
| **Model 2** | | | |
| 1. Restrained eating attitude | 22.55 ± 5.33 | 0.014 (−0.084 to 0.113) | 0.766 |
| 2. Anorexic attitudes | 20.23 ± 5.79 | −0.057 (−0.149 to 0.035) | 0.224 |
| 3&4. Cycle of bulimic crises | 30.32 ± 6.76 | 0.073 (−0.008 to 0.153) | 0.076 |
| 5. Impact of others’ perception | 10.41 ± 3.17 | −0.289 (−0.456 to −0.121) | **<0.001** |
| 6. Choice of food quality and time spent on meals | 7.44 ± 2.45 | −0.153 (−0.467 to 0.060) | 0.160 |
| **Model 3** | | | |
| 1. Restrained eating attitude | 22.53 ± 5.33 | 0.014 (−0.085 to 0.113) | 0.785 |
| 2. Anorexic attitudes | 20.23 ± 5.79 | −0.057 (−0.149 to 0.036) | 0.230 |
| 3&4. Cycle of bulimic crises | 30.32 ± 6.76 | 0.073 (−0.008 to 0.153) | 0.076 |
| 5. Impact of others’ perception | 10.41 ± 3.17 | −0.289 (−0.456 to −0.121) | ***<0.001*** |
| 6. Choice of food quality and time spent on meals | 7.44 ± 2.45 | −0.153 (−0.367 to 0.061) | 0.160 |

Data are presented as mean ± standard deviation (SD) for eating attitude subscale scores and as beta coefficients (β) with 95% confidence intervals derived from linear regression models. Beta coefficients represent the mean change in BMI per one-unit increase in the eating attitude subscale score. Model 2 was adjusted for age, sex, monthly income, and education level. Model 3 was additionally adjusted for medication use.
